# Localizing Synergies of Hidden Factors in Complex Systems: Resting Brain Networks and HeLa GeneExpression Profile as Case Studies

**DOI:** 10.3390/e27080820

**Published:** 2025-08-01

**Authors:** Marlis Ontivero-Ortega, Gorana Mijatovic, Luca Faes, Fernando E. Rosas, Daniele Marinazzo, Sebastiano Stramaglia

**Affiliations:** 1 INFN, Sezione di Bari, Dipartimento Interateneo di Fisica, Università degli Studi di Bari Aldo Moro, 70126 Bari, Italy; marlis.ontiveroortega@uniba.it; 2 Cuban Center for Neuroscience, Havana 53-72637112, Cuba; 3 Faculty of Technical Sciences, University of Novi Sad, 21000 Novi Sad, Serbia; gorana86@uns.ac.rs (G.M.); luca.faes@community.unipa.it (L.F.); 4 Dipartimento di Ingegneria, Università di Palermo, 90128 Palermo, Italy; 5 Department of Informatics, Center for Consciousness Science, Sussex AI, University of Sussex, Brighton BN1 9RH, UK; f.rosas@sussex.ac.uk; 6 Center for Psychedelic Research, Centre for Complexity Science, Department of Brain Science, Imperial College London, London SW7 2AZ, UK; 7 Center for Eudaimonia and Human Flourishing, University of Oxford, Oxford OX1 2JD, UK; 8 Department of Data Analysis, Ghent University, 9000 Ghent, Belgium; daniele.marinazzo@ugent.be

**Keywords:** latent factors, high order interactions, synergy, resting-state fMRI, cell gene expression

## Abstract

Factor analysis is a well-known statistical method to describe the variability of observed variables in terms of a smaller number of unobserved latent variables called factors. Even though latent factors are conceptually independent of each other, their influence on the observed variables is often joint and synergistic. We propose to quantify the synergy of the joint influence of factors on the observed variables using O-information, a recently introduced metric to assess high-order dependencies in complex systems; in the proposed framework, latent factors and observed variables are jointly analyzed in terms of their joint informational character. Two case studies are reported: analyzing resting fMRI data, we find that DMN and FP networks show the highest synergy, consistent with their crucial role in higher cognitive functions; concerning HeLa cells, we find that the most synergistic gene is STK-12 (AURKB), suggesting that this gene is involved in controlling the HeLa cell cycle. We believe that our approach, representing a bridge between factor analysis and the field of high-order interactions, will find wide application across several domains.

## 1. Introduction

Utilizing latent variables to represent datasets provides a robust approach for identifying the hidden structure and underlying drivers of complex systems, thereby enhancing data manageability and interpretability. Factor analysis (FA) [1] is a powerful statistical method widely used in a variety of fields, including behavioral sciences [2], social sciences [3], life sciences [4], physical sciences [5] and business [6], to describe variability among observed variables in terms of a smaller number of unobserved latent variables called factors. Specifically, observed variables are modeled as linear combinations of factors, short of some error or noise terms, and this method is extremely effective in reducing the set of relevant variables in large datasets. Factor analysis is closely related to principal component analysis [7] and therefore to singular value decomposition [8]. Principal component analysis entails a rotation of variable space in order to capture the maximum variance from the new variables. The three methods become identical when the error terms, or equivalently the variability not explained by the common factors, all have the same variance [9].

**Figure 1:**
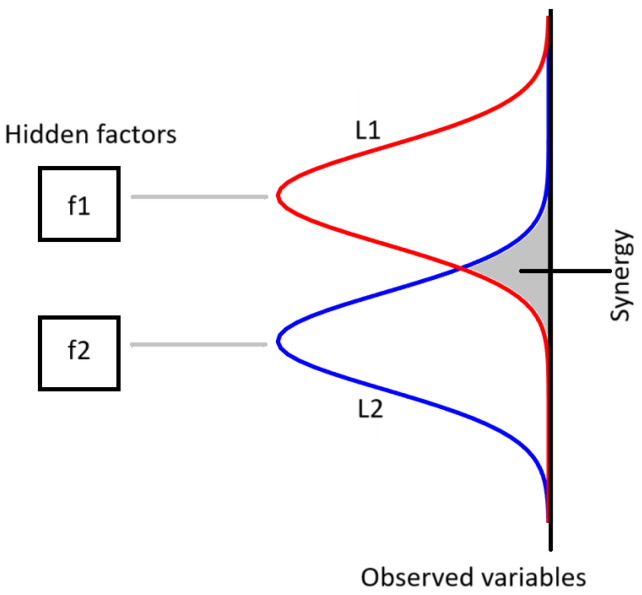
Diagram intuitively representing the proposed approach. Two hidden factors influence the observed variables: the overlap between the two loading profiles $L_{1}$ and $L_{2}$ corresponds to variables which are synergistic for factors $f_{1}$ and $f_{2}$.

Interestingly, even though latent variables derived from factor analysis (FA) are uncorrelated by design, they cooperate to produce effects on the observed variables. This makes it valuable to localize, within the complex system, those observed variables whose behavior relies on several factors, so we can quantify the synergistic impact of these factors. Intuitively we expect that such variables, whose role is to integrate the information from several factors, are those depending on more than one factor, as illustrated in Figure 1, where two factors influence the observed variables: the observed variables which depend on both factors are those encoding the joint action of the two factors, thus embodying the cooperation of the two latent variables. This work aims to quantify the cooperative effects of latent factors by using the concept of synergy, a notion recently developed within the framework of partial information decomposition [10] to identify variables responsible for integration in complex systems. In particular, we propose using the O-information metrics [11] to quantify cooperation and localize synergistic effects in the complex system. We refer the reader to [12] for a deep overview of the informational architecture of complex systems and to [13] for a discussion about biases in O-information estimation. While another method for localizing higher-order effects in complex systems, detailed in [14], utilizes gradients of O-information on groups of observed variables, our distinctive approach introduces latent variables to discard redundancy. This ensures that the net impact of these latent variables on the observed variables is solely synergistic.

## 2. Methods

Let us recall the definition of O-information [11], a metric measuring the balance between redundancy and synergy, the two basic types of high-order statistical dependencies. Let $x=\{x_{1},\ldots ,x_{n}\}$ denote a set of *n* stochastic variables, and let $x_{-i}$ denote the set of all the variables in x but $x_{i}$. The O-information is defined as [11]:

(1) $$O_{inf}\left(x\right)=\left(n-2\right)H\left(x\right)+\sum_{i=1}^{n}\left[H\left(x_{i}\right)-H\left(x_{-i}\right)\right],$$

where *H* is the Shannon entropy:

H(x)=−∫dxp(x)logp(x),

p(x) being the probability density function of x.

If $O_{inf}>0$, the system is redundancy-dominated. On the other hand, when $O_{inf}<0$, the dependencies are better explained as patterns that can be observed in the joint state of multiple variables but not in subsets of these; in other words, the system is synergy-dominated.

Now, we recall the generative model for FA, i.e.:

$$x=L\;f+\underline{\eta },$$

where x is the *N*-dimensional vector of observed variables, f is a vector of *m* latent factors, and L is the N×m matrix of loadings whilst $\underline{\eta }$ is a vector of *N* Gaussian noise terms with variances $\underline{\sigma }$. Factors are assumed to be Gaussian zero mean unit variance independent variables; factors and noise terms are uncorrelated. Given a suitable number of samples of x, both loadings and factor scores can be estimated by maximum likelihood.

For clarity, we concentrate now on the case of a group of latent factors influencing a single target variable. Let $f_{1},f_{2},\ldots ,f_{m}$ be *m* independent latent variables acting as drivers for the target

$$x=\sum_{i=1}^{m}L_{i}f_{i}+\eta ,$$

where η is a Gaussian noise term with variance $\sigma^{2}$. The structure corresponding to this equation is a collider, corresponding to net synergy. We recall that all fs are assumed to be zero mean unit variance Gaussian variables and that *x* has unit variance, i.e., $\sum_{i=1}^{m}L_{i}^{2}+\sigma^{2}=1.$ The mutual information between the observed variable *x* and factor $f_{i}$ is given by $I\left(x;f_{i}\right)=-\frac{1}{2}\log\left(1-L_{i}^{2}\right),$ with $I\left(x;f_{i}\right)∼\frac{1}{2}\;L_{i}^{2}$ for small $L_{i}$; it follows that the influence of factors on the observed variable is controlled by loadings $L_{i}$.

Now we turn to consider higher-order dependencies. Straightforward calculations, based on the formula for the entropy of a multivariate Gaussian distribution [15], provide the O-information for the group of n=m+1 variables $\left\{f_{1}\ldots ,f_{m},x\right\}$:

(2) $$O_{inf}=\frac{1}{2}\log\frac{{\left(1-\sum_{i=1}^{m}L_{i}^{2}\right)}^{m-1}}{\prod_{i=1}^{m}\left(1-\sum_{k\neq i}L_{k}^{2}\right)}$$

Note that this quantity is always less than zero or it vanishes, thus confirming the occurrence of net synergy. We remark that $O_{inf}$ is zero if all Ls vanish but one. For small Ls, $O_{inf}$ is the order

$$O_{inf}∼-\sum_{i\neq k}L_{i}^{2}L_{k}^{2}.$$

The formula above quantitatively supports the scenario described in Figure 1, i.e., an observed variable is synergistic for two latent factors only if it is dependent on both latent factors. In the Appendix A, we report a theorem which generalizes the non-positivity of $O_{inf}$, shown above for Gaussian variables, to the case of a general probability distribution, when a group of independent variables influence a target variable. This theorem is useful in applications where latent factors are inferred by Independent Component Analysis (ICA) [16].

The calculation above suggests the following procedure to highlight synergy: (i) fix the number of latent factors; (ii) fit the FA model to data and extract the latent factor scores; (iii) for each observed variable, evaluate $O_{inf}$ for the multiplet constituted by the observed variable plus the latent factors $-O_{inf}$ representing the net synergy; and (iv) the variables with the most negative values of $O_{inf}$ are the most involved in synergistic behavior; in other words, they are responsible for integration in the system.

We remark that to evaluate $-O_{inf}$ one may either (i) take the estimated loading matrices and use Equation (2), or (ii) perform a direct evaluation by firstly estimating samples of factors, then evaluating the $O_{inf}$ of factors and the measured variables using (1); in this work, we used (ii).

## 3. Results

In the following section, we describe two applications of the proposed framework in biomedical engineering, i.e., the analysis of resting-state fMRI time series of healthy subjects, and the analysis of gene expression data from HeLa cells.

### 3.1. fMRI Data

We use the public dataset described in Poldrack et al. [17]. This dataset was obtained from the OpenfMRI database, with accession number ds000030, and was already used in [18]. We use resting-state fMRI data from 121 healthy controls and 152 time points. The demographics are reported in the original paper.

Data were preprocessed with FSL (FMRIB Software Library v5.0) [19]. The volumes were corrected for motion, after which slice timing correction was applied to correct for temporal alignment. All voxels were spatially smoothed with a 6 mm FWHM (full width at half maximum) isotropic Gaussian kernel and after intensity normalization, a band pass filter was applied between 0.01 and 0.08 Hz. In addition, linear and quadratic trends were removed. We next regressed out the motion time courses, the average cerebrospinal fluid signal, and the average white matter signal. Global signal regression was not performed. Data were transformed to the MNI152 template, such that a given voxel had a volume of 3 mm × 3 mm × 3 mm. Finally, we averaged the signal in 268 ROIs. In order to localize the results within the intrinsic connectivity network of the resting brain, we assigned each of these ROIs to one of the nine resting-state networks (seven cortical networks, plus subcortical regions and cerebellum) as described in [20]. Time series from subjects were firstly z-scored and then concatenated in a matrix with 268 observed variables and 152×121=18392 samples.

We applied the factor analysis function *factoran* of MATLAB to fit this matrix, with the number of hidden factors fixed at 20 (we found that results are robust to slight variations of the number of factors). Some factors thus obtained were recognized to be related to common trends of data; therefore, we removed from the subsequent analysis 3 factors mostly correlated to the common trend, and we were left with 17 factors.

Then, for each region, we evaluated the O-information of that region and the 17 latent factors; the statistical significance of $O_{inf}$ can be assessed using surrogates obtained by random permutation of the samples of the target region.

**Figure 2:**
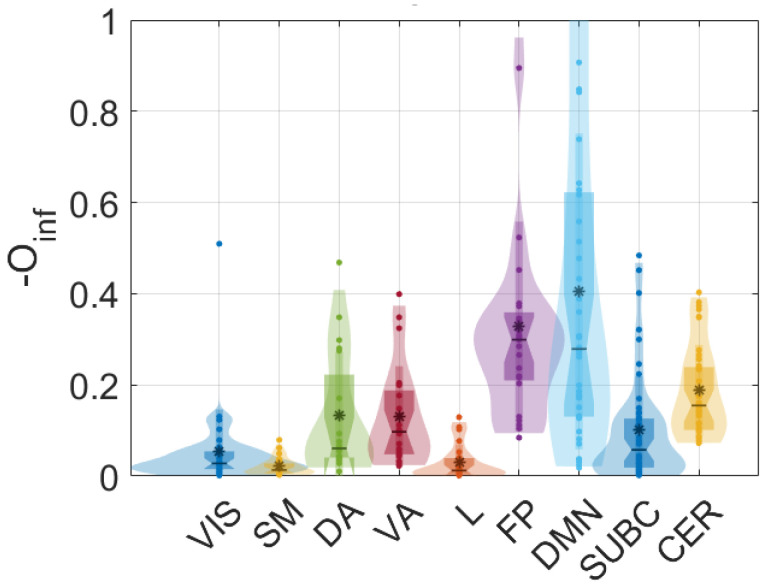
The opposite of the O-information (measuring net synergy) is plotted for the 268 regions in the fMRI dataset.

The results are shown in Figure 2. We find that FP and DMN show the highest synergy, and that generically synergy is higher in associative areas supporting cognitive functions and lower in sensory-motor areas, consistent with previous analyses which use other tools to evaluate higher-order dependencies [21,22,23].

### 3.2. HeLa Data

We apply the proposed approach to data from the cell culture HeLa. The data correspond to 94 genes and 48 time points [24], with an hour interval separating two successive readings (the HeLa cell cycle lasts 16 h). The 94 genes were selected, from the full dataset described in [25], on the basis of the association with cell cycle regulation and tumor development. Since the number of samples is less than the number of variables, in this case we use principal component analysis instead of FA. As described in [26], the first two principal components show exponentially decaying correlations whilst the third principal component seems to be connected with cell cycle as it shows oscillations with a period close to 16. We discard the first principal component and consider here the second and the third components to find the genes which exhibit synergy with regard to these two factors.

**Figure 3:**
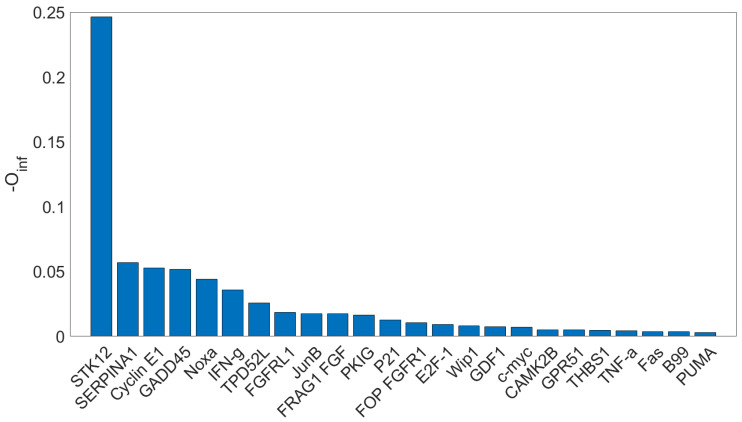
The opposite of the O-information (measuring net synergy) is plotted for the 24 genes in the HeLa dataset whose synergy is recognized as statistically significant against surrogates after Bonferroni correction. The highest synergy is obtained in the correspondence of the gene STK12.

The results are depicted in Figure 3; 24 genes, out of 94, show a synergy which is statistically higher than those from surrogates after Bonferroni correction. The highest synergy is obtained in correspondence of STK-12 (Aurora Kinase B), which is known to work as a transcriptional brake, controlling the expression of genes involved in cellulase production [27]. A synergistic interaction between Aurora B and ZAK has been found in triple-negative breast cancer [28]: our analysis shows that it may also play a key role also in the control of the HeLa cell cycle.

## 4. Discussion

Many complex systems can be effectively described by latent factors that influence observable variables: this representation helps eliminating data redundancy, so as to reveal synergies that might otherwise be hidden. In this work, we introduce a novel perspective on analyzing higher-order dependencies. We propose evaluating the informational characteristics of circuits that include both latent factors and observed variables. This method allows us to pinpoint specific variables within the system whose behavior synergistically depends on these factors, thus identifying them as information integrators. Specifically, we suggest using O-information to assess the informational character of groups of variables comprising the latent factors and each individual observed variable. This enables us to quantify the synergistic role played by each observed variable.

We apply the proposed methodology to fMRI data from healthy individuals, and found less synergy in sensory networks with regard to networks which support complex cognitive processes such as planning and execution of goal-directed behavior [29]. Applying our method to genetic data from HeLa cells, we found that the most synergistic gene is STK-12, whose synergistic role was already assessed by performing an analysis at the level of observed gene expressions.

We believe this approach can offer further insights into complex systems that lend themselves to a suitable representation using latent variables.

Further research will focus on integrating our proposed approach with other methods for inferring latent factors, such as ICA, as well as tackling the crucial problem of selecting the most appropriate factors for analysis. Indeed, choosing the optimal number of factors can significantly impact the results; there is no single perfect method to fix it, and instead, it is often a combination of statistical criteria, theoretical considerations, and practical judgment. We remark that increasing the number of factors cannot decrease the synergy of any observed variable, according to (2); however, the relative synergy of two observed variables may change. The topic of further investigation will be the search for a protocol for the suitable number of latent variables to measure synergies. Another interesting area of study will be to explore the relationship between the synergies of factors (as introduced here) and those measurable on groups of observed variables. This essentially means investigating the connection between underlying mechanisms and emergent behaviors within this context [30].

## Data Availability

The data presented in this study are available in https://openfmri.org accessed on 28 May 2025.

## Appendix A

Here we prove a theorem about the non-positivity of the O-information, for arbitrary probability distributions, when a group of independent variables influence a target variable. Consider *n* random variables $X^{n}=\left(X_{1},\ldots ,X_{n}\right)$ that follow a joint distribution with the following structure: $p\left(x\right)=p\left(x_{n}|x^{n-1}\right)\prod_{i=1}^{n-1}p\left(x_{i}\right)$.

**Statement** **1.** $O_{inf}\left(X^{n}\right)\leq 0$.

**Proof.** Firstly we recall that O-information is the difference between the total correlation (TC) and the dual total correlation (DTC) [11], i.e., $O_{inf}\left(X^{n}\right)=TC\left(X^{n}\right)-DTC\left(X^{n}\right)$. It can be shown that

(A1) $$\begin{array}{cc} \mathrm{TC}(X^{n}) & =\sum_{j=2}^{n}I\left(X^{j-1};X_{j}\right), \end{array}$$

(A2) $$\begin{array}{cc} \mathrm{DTC}(X^{n}) & =I\left(X^{n-1};X_{n}\right)+\sum_{k=2}^{n-1}I\left(X^{k-1};X_{k}|X_{k+1}^{n}\right), \end{array}$$

where $X_{i}^{j}=\left(X_{i},\ldots ,X_{j}\right)$ is a shorthand notation. Then, because of the joint distribution of $X^{n}$, it is clear that

(A3) $$I(X^{i-1};X_{i})=0$$

for all i<n. This implies that, for this particular type of joint distribution, we have

(A4) $$\mathrm{TC}\left(X^{n}\right)=I\left(X^{n-1};X_{n}\right).$$

Note that this is the first term in the expression for the DTC above, and hence this implies $TC\left(X^{n}\right)\leq DTC\left(X^{n}\right)$ for this type of distribution. Moreover, we can represent the value of the O-information in this case as follows:

(A5) $$O_{inf}\left(X^{n}\right)=\mathrm{TC}\left(X^{n}\right)-\mathrm{DTC}\left(X^{n}\right)=-\sum_{k=2}^{n-1}I\left(X^{k-1};X_{k}|X_{k+1}^{n}\right)\leq 0,$$

which proves the statement. □
